# Combination Treatment of Deep Sea Water and Fucoidan Attenuates High Glucose-Induced Insulin-Resistance in HepG2 Hepatocytes

**DOI:** 10.3390/md16020048

**Published:** 2018-02-02

**Authors:** Shan He, Wei-Bing Peng, Hong-Lei Zhou

**Affiliations:** 1 School of Pharmacology, Shandong University of Traditional Chinese Medicine, Jinan 250355, China; zhouhongleitcm@163.com; 2 Biology Institute, Qilu University of Technology (Shandong Academy of Sciences), Jinan 250103, China; weibingpeng@hotmail.com

**Keywords:** deep-sea water, fucoidan, IR-HepG2, Akt/GSK-3β pathway, AMPK-ACC pathway

## Abstract

Insulin resistance (IR) plays a central role in the development of several metabolic diseases, which leads to increased morbidity and mortality rates, in addition to soaring health-care costs. Deep sea water (DSW) and fucoidans (FPS) have drawn much attention in recent years because of their potential medical and pharmaceutical applications. This study investigated the effects and mechanisms of combination treatment of DSW and FPS in improving IR in HepG2 hepatocytes induced by a high glucose concentration. The results elucidated that co-treatment with DSW and FPS could synergistically repress hepatic glucose production and increase the glycogen level in IR-HepG2 cells. In addition, they stimulated the phosphorylation levels of the components of the insulin signaling pathway, including tyrosine phosphorylation of IRS-1, and serine phosphorylation of Akt and GSK-3β. Furthermore, they increased the phosphorylation of AMPK and ACC, which in turn decreased the intracellular triglyceride level. Taken together, these results suggested that co-treatment with DSW and FPS had a greater improving effect than DSW or FPS alone on IR. They might attenuate IR by targeting Akt/GSK-3β and AMPK pathways. These results may have some implications in the treatment of metabolic diseases.

## 1. Introduction

Recently, changes in life styles and eating habits have resulted in so-called lifestyle-related illnesses such as hyperlipidemia, hypertension, and diabetes, leading to increased morbidity and mortality rates and a social burden worldwide. Most of these diseases are characterized by one distinct defect: insulin resistance (IR) [1]. IR is defined as a diminished ability of some kinds of cells, such as adipocytes, skeletal muscle cells, and hepatocytes, to respond to the action of insulin, which plays a central role in the development of several metabolic abnormalities and diseases, such as obesity, type 2 diabetes, and metabolic syndrome. 

Many studies have suggested that the defect of insulin signaling is the main reason for IR. The liver is an insulin sensitive organ that plays a key role in the regulation of whole body energy homeostasis, and hepatic IR immensely increases the risk of impaired fasting glucose and type 2 diabetes [2]. It has been shown that human HepG2 hepatoma cells are a suitable cell model in insulin signaling investigations [3]. Besides, the high glucose condition causes a significant increase of the serine (Ser) 307 phosphorylation level of insulin receptor substrate-1 (IRS-1) in HepG2 cells, leading to reduced insulin-stimulated phosphorylation of Akt. As a result of this, the metabolic effects of insulin on glycogen synthesis and glucose uptake are inhibited by a high glucose level [4]. Therefore, a stable IR cell model can be established using HepG2 cells treated with a high concentration of glucose.

The AMP-activated protein kinase (AMPK) system acts as a sensor of cellular energy status and it is a key player in the development and treatments of obesity, type 2 diabetes, and metabolic syndrome [5]. The AMPK phosphorylation level in threonine (Thr) 172 is currently accepted as a marker of AMPK activity. Once activated, AMPK switches on catabolic pathways that generate ATP, while switching off ATP-consuming processes. Moreover, previous research elucidated that activated AMPK rapidly phosphorylated IRS-1 on Ser789, which led to an increase in insulin-stimulated IRS-1-associated PI3K activity [6].

Although many antidiabetic drugs have been put on the market in recent years, most of them can cause significant side effects and tolerability problems [7]. Recently, as an alternative strategy for developing more effective and safe drugs, many natural products, including crude extracts isolated from natural resources, are being investigated to treat metabolic diseases [8]. 

Deep sea water (DSW) generally refers to sea water from a depth of more than 200 m. DSW is a safe and stable natural resource, which is in infinite supply compared with other natural products. It contains high levels of minerals such as magnesium (Mg) and calcium (Ca), as well as several beneficial trace elements such as zinc (Zn), manganese (Mn), vanadium (V), chromium (Cr), and selenium (Se). Furthermore, several studies demonstrated that DSW exerted diverse biological activities, such as regulation of the immune system and antioxidant activity [9], preventive and therapeutic effects on cardiovascular diseases [10,11,12,13,14] and diabetes mellitus [15,16,17,18], and an antifatigue effect [19]. In vivo and in vitro studies showed that DSW of hardness 1000 ppm exerted anti-obesity, anti-hyperlipidemia, and anti-diabetic properties, including the inhibition of adipocyte differentiation and lipid accumulation, as well as improving impaired glucose tolerance [11,12,13,14,15,16,17]. In addition, DSW of hardness 1000 ppm showed no acute or subacute effect [20]. Therefore, it is worth studying the possibility that DSW of hardness 1000 ppm might be an eye-catching agent for treating or preventing metabolic disease.

Fucoidans (FPS) are fucose-rich polysaccharides isolated from brown alga, which have been proved to exhibit a wide spectrum of biological activities, such as anticoagulation, anti-tumor, antioxidant, immune regulation, antiviral, and anti-inflammatory activity, as well as protection of the liver, kidney, and urinary system [21,22]. FPS are abundant cost-effective marine resources which have been investigated in recent years to develop novel drugs and functional foods [23].

It is worth noting that administering lower doses of two agents in combination may be more efficacious than higher or maximal doses of a single agent. Furthermore, such therapy can avoid the risk of adverse events due to higher doses in monotherapy [24]. DSW and FPS are two types of natural resources with little side effects, and different active ingredients and modes of action, which could be taken into account as a novel agent in combination therapy for the prevention or treatment of metabolic diseases.

Accordingly, this study investigated whether combination treatment of DSW and FPS could modulate IR effectively in HepG2 hepatocytes induced by a high glucose concentration and thus developed a new understanding of its action mechanisms.

## 2. Results

### 2.1. Analysis of Element Contents in DSW of Hardness 1000 ppm

Element contents in DSW of hardness 1000 ppm are shown in Table 1. The data show that, in terms of macro elements, DSW had an Na:Ca:Mg ratio of 1:1:3. Besides, DSW was rich in essential trace elements, such as V, Cr, Mn, Zn, and Se, while harmful trace elements such as mercury (Hg) and plumbum (Pb) were not detected (ND) in DSW. 

### 2.2. Analysis of FPS Physicochemical Property

**Figure 1:**
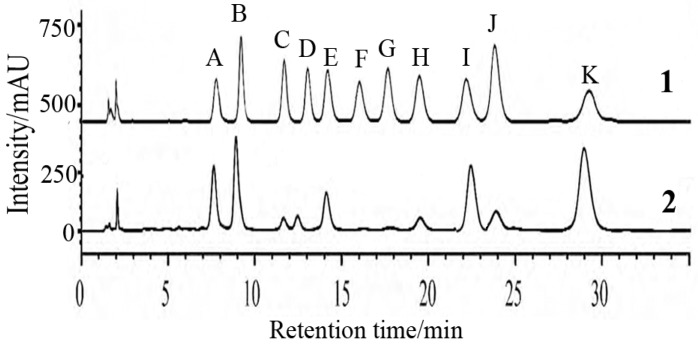
HPLC chromatogram of FPS extracted from *Sargassum pallidum*. Note: A. PMP; B. d-mannose (Man); C. d-glucosamine (GlcN); D. l-rhamnose (Rha); E. d-glucruonic acid (GlcA); F. d-galacturonic acid (GalA); G. d-galactosamine (GalN); H. d-glucose (Glc); I. d-galactose(Gal); J. d-xylose (Xyl); K. l-fucose (Fuc). 1-Monosaccharide standards; 2-FPS extracted from *Sargassum pallidum*.

The monosaccharide composition in FPS was analyzed by high performance liquid chromatography (HPLC). Analysis of Figure 1 showed that FPS were mostly composed of fucose (51.4%), mannose (24.3%), and galactose (20.6%). The contents of total carbohydrates and the sulfate group were 73.6% and 10.9%, respectively.

### 2.3. Cytotoxity of Combination Treatment of DSW and FPS on Hepg2 Cells

Previously, in vitro studies suggested that DSW of hardness 1000 ppm had no effect on cell viability, including HepG2 cells and 3T3-L1 Adipocytes [11,17]. Meanwhile, it also showed no acute or subacute effect in vivo, which did not affect hematological parameters, blood biochemical parameters, or the morphology of the main internal organs, such as the liver, kidney, and pancreas [20]. How about the safety of DSW of hardness 1000 ppm combined with FPS?

**Figure 2:**
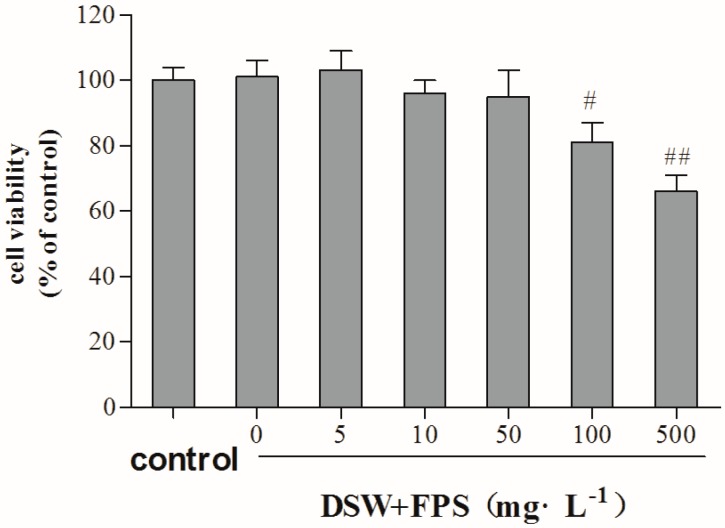
Cytotoxity of combination treatment of DSW and FPS on HepG2 cells. HepG2 cells were incubated with co-treatment with DSW and FPS of different concentrations (5, 10, 50, 100, and 500 mg·L^−1^) for 24 h. Then, cell viability was measured by the MTT method. Data are presented as mean ± SD from three independent experiments (*n* = 8 per group). ^#^ *p* < 0.05, ^##^ *p* < 0.01: Significant difference vs. the control group.

We initially examined the cytotoxicity of co-treatment with DSW and FPS on HepG2 cells using the MTT assay. Data in Figure 2 show that co-treatment with DSW and FPS at concentrations below 50 mg·L^−1^ had no significant influence on the viability of HepG2 cells. However, incubation of DSW and FPS at 100 mg·L^−1^ or higher concentrations significantly decreased the cell viability compared with the control group. Accordingly, FPS at 50 mg·L^−1^ was considered as the maximum non-toxic combination dose in our experiments, and the combination doses of FPS used in the following experiments were settled as: 5, 10, 20, and 50 mg·L^−1^.

### 2.4. Combination Treatment of DSW and FPS Repressed Hepatic Glucose Production and Increased Glycogen Level in IR-Hepg2 Cells

**Figure 3:**
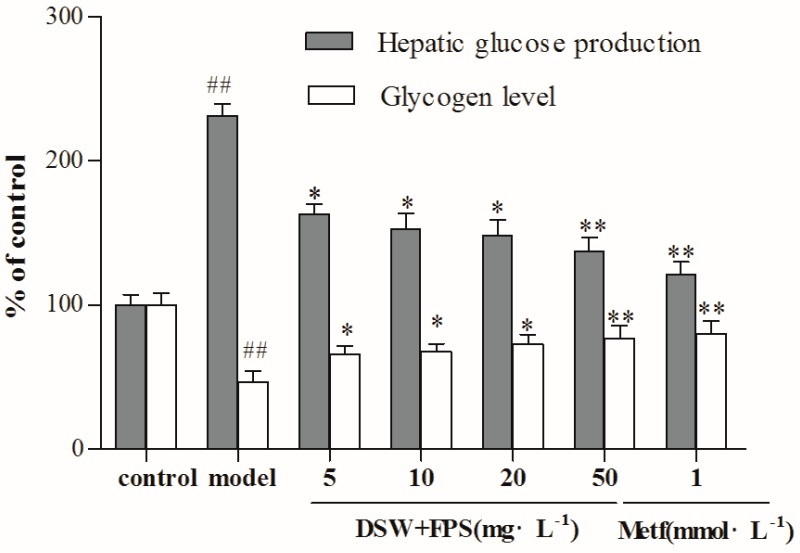
Combination treatment of DSW and FPS repressed hepatic glucose production and an increased glycogen level in IR-HepG2 cells. HepG2 cells were co-treated with a high glucose concentration and combination of DSW and FPS of different concentrations (5, 10, 20, and 50 mg·L^−1^) for 24 h. Then, levels of hepatic glucose production and glycogen were detected using kits respectively. Data were presented as mean ± SD from three independent experiments (*n* = 8 per group). ^##^ *p* < 0.01: Significant difference vs. the control group; * *p* < 0.05, ** *p* < 0.01: Significant difference vs. the model group.

As shown in Figure 3, the exposure of human HepG2 hepatocytes to a high glucose concentration (30 mmol·L^−1^) for 24 h decreased intracellular glycogen content and increased gluconeogenesis in the presence of 1 nmol·L^−1^ insulin, which suggested that the role of insulin in terms of inhibiting hepatic glucose production was impaired. However, combination treatment of DSW and FPS of different concentrations (5, 10, 20, and 50 mg·L^−1^) decreased the hepatic glucose production and increased the glycogen level in IR-HepG2 cells in a dose-dependent manner, which implied that co-treatment with DSW and FPS synergistically improved the insulin sensitivity of IR-HepG2 cells. Therefore, FPS at 50 mg·L^−1^ is the most effective combination dose.

### 2.5. Comparison on Hepatic Glucose Production and Glycogen Level between Combination Treatment of DSW and FPS and Single Application of DSW or FPS in IR-HepG2 

**Figure 4:**
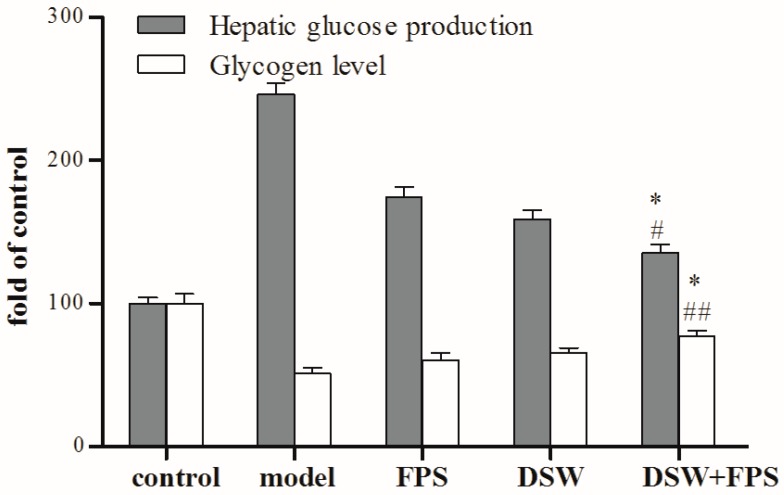
Comparison of hepatic glucose production and glycogen level between combination treatment of DSW and FPS and single application of DSW or FPS in IR-HepG2. HepG2 cells were co-treated with a high glucose concentration and DSW alone, or FPS alone, or a combination of DSW and FPS at a dosage of 50 mg·L^−1^ for 24 h. Then, levels of hepatic glucose production and glycogen were detected using kits respectively. Data were presented as mean ± SD from three independent experiments (*n* = 8 per group). ^#^ *p* < 0.05, ^##^ *p* < 0.01: Significant difference vs. the FPS group; * *p* < 0.05,: Significant difference vs. the DSW group.

We next determined whether combination treatment of DSW and FPS at a dosage of 50 mg·L^−1^ was more efficient in inhibiting hepatic glucose production compared with the single application of DSW or FPS. Data showed that, compared with the single use of DSW or FPS, combination treatment of DSW and FPS at a dosage of 50 mg·L^−1^ exerted higher potency to decrease the hepatic glucose production and enhance glycogen synthesis in IR-HepG2 cells, as presented in Figure 4. Thereafter, we further elucidated the mechanisms of action of its insulin-enhancing effect in IR-HepG2 cells.

### 2.6. Effects of Combination Treatment of DSW and FPS on Insulin Signaling in HepG2 Cells under High Glucose Condition

To further clarify the regulatory mechanism by which co-treatment with DSW and FPS inhibited the hepatic glucose production in IR-HepG2 cells, we investigated the expression and phosphorylation levels of components in the insulin signaling pathway by western blot analysis.

**Figure 5:**
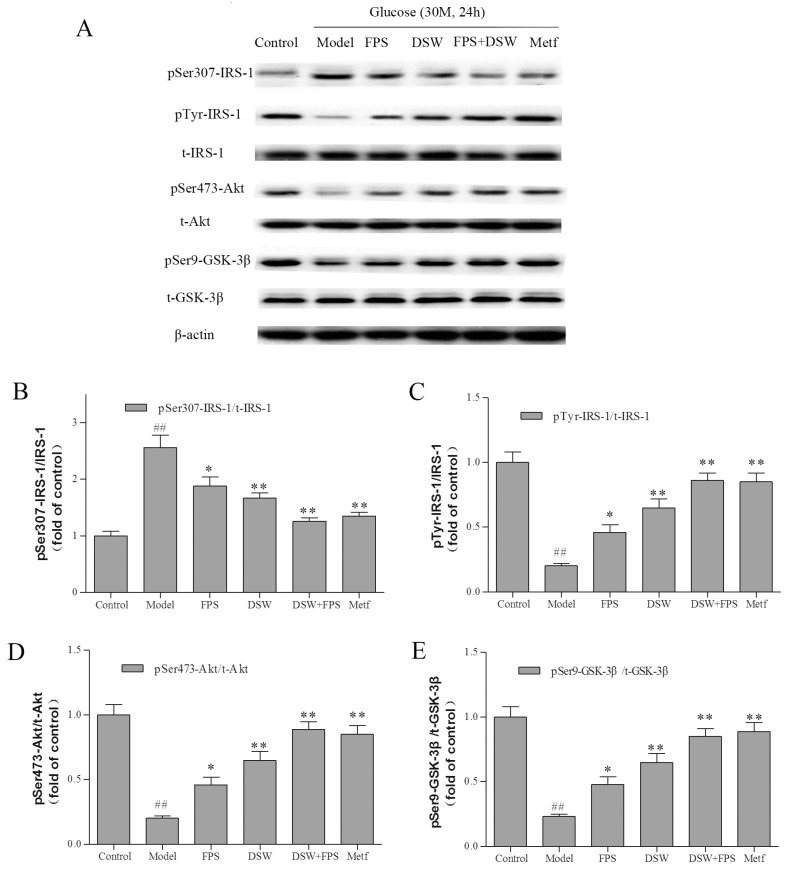
Combination treatment of DSW and FPS synergistically stimulated the Akt/GSK-3β signaling in HepG2 cells under a high glucose condition. Cells were treated with DSW and/or FPS under a high glucose concentration for 24 h. At the end point of co-treatment, cells were stimulated by 100 nmol·L^−1^ insulin for 10 min and then harvested. (**A**) The expression and phosphorylation levels of IRS-1, Akt, and GSK-3β were detected by western blot, and β-actin was used as a loading control. The phosphorylation levels of IRS-1 (**B**,**C**), Akt (**D**) and GSK-3β (**E**) were presented as mean ± SD from three independent experiments (*n* = 3 per group), and the figures were the most representative. ^##^ *p* < 0.01: Significant difference vs. the control group; * *p* < 0.05, ** *p* < 0.01: Significant difference vs. the model group.

Long-term exposure to glucose has been found to induce IR through reducing the IRS sensitivity and resulted in the blockade of insulin signaling [25]. As shown in Figure 5, insulin-induced tyrosine (Tyr) phosphorylation of insulin receptor substrate-1(IRS-1) took place normally under a normal concentration (5.5 mmol·L^−1^) of glucose treatment. However, the high glucose condition caused significant increasing Ser 307 phosphorylation of IRS-1, while the insulin-stimulated Tyr phosphorylations of IRS-1 was significantly decreased, leading to reduced insulin-stimulated Ser 473 phosphorylation of Akt. In parallel, Ser 9 phosphorylation of glycogen synthase kinase-3β (GSK-3β) in HepG2 cells was also clearly blocked. This phenomenon was consistent with the increased hepatic glucose production and reduced glycogen level mentioned above. Besides, the total protein levels of IRS-1, Akt, and GSK-3β did not significantly alter after high glucose treatment.

In contrast, both FPS and DSW could stimulate Akt/GSK-3β signaling through enhancing Tyr phosphorylation of IRS-1 and Ser phosphorylation of Akt and GSK-3β. Meanwhile, the decreased phosphorylation levels of IRS-1, Akt, and GSK-3β could also be synergistically reversed by co-treatment with DSW and FPS, which was in line with the sharply decreased hepatic glucose production level in this group, as shown in Figure 4.

### 2.7. Combination Treatment of DSW and FPS Reversed the Suppression of AMPK and ACC Phosphorylation and the Accumulation of Triglyceride by High Glucose Concentration 

Triglyceride is a central feature of IR. It has been shown that triglyceride could cause IR through serine phosphorylation of IRS-1 that, in turn, lead to the suppression of insulin receptor signaling through Tyr dephosphorylation of IRS-1 [26]. Acetyl-CoA carboxylase (ACC), which plays a pivotal role in hepatic lipid metabolism, is controlled by allosteric regulation by citrate and glutamate and by covalent modification by phosphorylation, and AMPK inhibits ACC by phosphorylation of Ser 79 [27].

**Figure 6:**
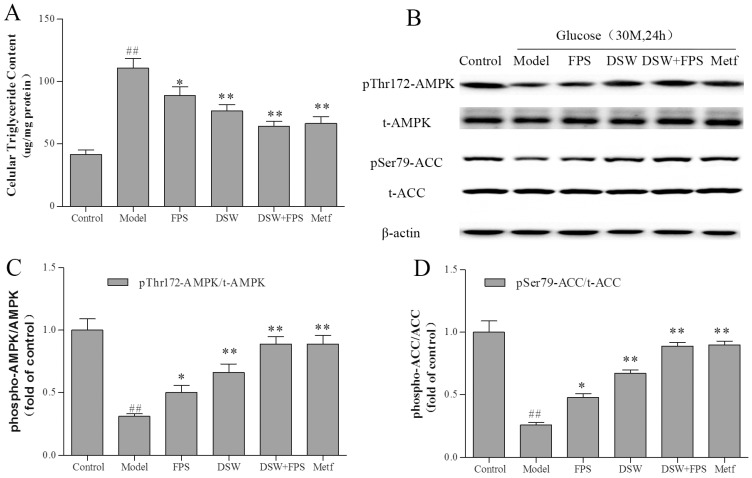
Combination treatment of DSW and FPS inhibited cellular triglyceride accumulation and stimulated AMPK and its targets in HepG2 cells under a high glucose condition. Cells were treated with DSW and/or FPS under a high glucose concentration for 24 h. (**A**) Levels of intracellular triglyceride in cells were measured using spectrophotometric assays and expressed as μg/mg of protein; (**B**) The expression and phosphorylation levels of AMPK and ACC were detected by western blot, and β-actin was used as a loading control. The phosphorylation levels of AMPK (**C**) and ACC (**D**) were presented as mean ± SD from three independent experiments (*n* = 3 per group), and the figures were the most representative. ^##^ *p* < 0.01: Significant difference vs. the control group; * *p*< 0.05, ** *p* < 0.01: Significant difference vs. the model group.

To better understand the role in the regulation of hepatic lipid metabolism, we studied the effect of co-treatment with DSW and FPS on AMPK and its downstream effector, ACC, as well as on triglyceride content in HepG2 cells. As shown in Figure 6, exposure of HepG2 cells to high glucose (30 mmol·L^−1^, 24 h) decreased the phosphorylation of AMPK and ACC without an obvious change in total protein levels (Figure 6B–D). In concert with these changes in the phosphorylation of AMPK and ACC, triglyceride content dramatically increased by almost three-fold in IR hepatocytes (Figure 6A). On the contrary, combination treatment of DSW and FPS markedly up-regulated the phosphorylation levels of AMPK and ACC. Moreover, the intracellular contents of triglyceride were also lowered by 40%.

## 3. Discussion

This study investigated the effects and mechanisms of combination treatment of DSW and FPS in improving IR in HepG2 hepatocytes induced by a high glucose concentration.

The results suggested that co-treatment with DSW and FPS at different concentrations (5, 10, 20, and 50 mg·L^−1^) decreased hepatic glucose production and increased the glycogen level in IR-HepG2 cells in a dose-dependent manner. Moreover, compared with the single use of DSW or FPS, combination treatment of DSW and FPS at a dosage of 50 mg·L^−1^ synergistically decreased hepatic glucose production and enhanced glycogen synthesis in IR-HepG2 cells. Thereafter, we further elucidated the mechanisms of action of its insulin-enhancing effect in IR-HepG2 cells.

In hepatocytes, IR can result from impaired signaling downstream of the insulin receptor [28]. Tyr phosphorylation of IRS by insulin is a crucial event in mediating insulin action, defective in most cases of IR, both in experimental models and in humans. Akt is the key molecule which mediates the metabolic effects of insulin signaling. It lays downstream of phosphatidylinositol 3-kinase (PI-3K). Activated Akt induces glycogen synthesis through the inhibition of GSK-3 and prevents the liver from producing more glucose by the inhibition of glycogenolysis and gluconeogenesis. 

Combination treatment of DSW and FPS displays insulin-enhancing effects, but are these effects associated with the activation of insulin signaling? The cellular proteins and phosphorylation levels of IRS-1, Akt, and GSK-3β in total cell lysates were evaluated by western blot analysis. The data showed that long-term exposure to glucose induced IR through reducing the IRS sensitivity and resulted in the blockade of insulin signaling. In contrast, co-treatment with DSW and FPS could stimulate the IRS-1 signaling through enhancing its Tyr phosphorylation after 24 h under a high glucose condition. Meanwhile, the inhibition of phosphorylation Akt and GSK-3β could also be synergistically reversed by co-treatment with DSW and FPS.

AMPK activation is thought to be a key proximal event in the cellular energy balance response, and triglyceride is a central feature of IR. We further investigated the effect of co-treatment with DSW and FPS on AMPK and its downstream effector, ACC, as well as on triglyceride content in HepG2 cells. The results indicated that the inhibition of AMPK and ACC phosphorylation in cells exposed to high glucose concentrations was synergistically restored by co-treatment with DSW and FPS, which was consistent with a decreasing intracellular triglyceride level. 

Taken together, the present study suggested that IR induced by a high glucose concentration in HepG2 hepatocytes could be improved by combination treatment of DSW and FPS. They could synergistically repress hepatic glucose production and increase the glycogen level. Besides, they stimulated the Tyr phosphorylation of IRS-1, in addition to the phosphorylation of Akt and GSK-3β, which in turn decreased hepatic glucose production and enhanced glycogen synthesis. Moreover, they also activated the phosphorylation of AMPK and ACC, which in turn decreased the intracellular triglyceride level. In short, they might reverse IR in HepG2 cells by targeting Akt/GSK-3β and AMPK pathways.

DSW has been elucidated to activate the AMPK pathway in hyperlipidemia subjects [10,11] and diabetic mice [15,18]. These pharmacological activities must be due to the mineral elements contained in DSW. Many researches have shown that minerals in DSW are involved in insulin signaling. For example, intracellular Mg is a critical cofactor for more than 300 enzymes involved in carbohydrate and lipid metabolism, especially those involved in phosphorylation reactions such as Tyr kinase [29,30,31,32,33]. Intracellular Ca acts as a second messenger in many signal transduction pathways. Several reports indicate that intracellular Ca regulates insulin signaling, possibly due to the Ca^2+^ inhibition of insulin-regulated dephosphorylation. Moreover, the binding of Ca^2+^ to the plasma membrane may play important roles in insulin’s action on fat cell function [34,35]. Besides, Cr improves insulin binding, insulin internalization, and oxidative stress, as well as increases the number of insulin receptors with overall increases in insulin sensitivity [36,37,38,39]. Furthermore, Mg and Ca supplements could activate AMPK and deactivate ACC and HMG-CoA Reductase, lowering the levels of triglyceride and cholesterol [40,41].

FPS also could improve insulin sensitivity in vivo and vitro. Researchers have shown that low molecular weight FPS effectively ameliorated glucose homeostasis by elevating glucose tolerance and reduced lipid parameters in db/db mice. In addition, it could markedly reverse the reduced phosphorylation level of AMPK and Akt by ER stressors [42]. Besides, FPS ameliorate IR by suppressing oxidative stress and inflammatory cytokines in experimental non-alcoholic fatty liver disease [43]. FPS also stimulate insulin secretion and provide pancreatic protection via the cAMP signaling pathway, both in in vivo and in vitro studies [44].

To date, the synergistic effect of combination treatment of DSW and FPS on IR could be attributed to their stronger stimulation of Akt/GSK-3β and AMPK-ACC pathways, which might be associated with their different active ingredients and modes of action. Maybe, there are other pathways or targets involved in these actions. Future investigation on these matters and the in vivo study may provide new therapeutic approaches for the management of metabolic disease.

## 4. Materials and Methods

### 4.1. Materials 

DSW was pumped up from a depth of 1 km and a distance of 500 km off coastline of Shantou (120°30′15″ E, 20°59′57″ N, South China Sea) using a CTD water sampler (Sea-Bird Electronics Inc., Bellevue, DC, USA). Dulbecco’s modified Eagle’s medium (DMEM) and fetal bovin serum (FBS) were obtained from Gibco; insulin, and standard samples of monosaccharide, such as Fuc, Gal, Man, Xyl, Glc, and Xyl, were purchased from Sigma; the BCA protein assay kit was from the Beyotime Institute of Biotechnology (Shanghai, China); the glucose assay kit and TG assay kit were from Nanjing Jiancheng Bioengineering Institute (Nanjing, China); SDS-polyacrylamide gel electrophoresis was from Bio Rad, (Hercules, CA, USA); nitrocellulose membranes were from Amersham Biosciences (Uppsala, Sweden); antibodies against β-actin, IRS-1, phospho-IRS-1 (Ser307), Akt, phospho Akt (Ser473), GSK-3β, phospho-GSK-3β (Ser9), AMPKα, phosphor-AMPKα (Thr172), ACC, and phospho-ACC (Ser79) were purchased from Cell Signaling Technology (Beverly, MA, USA).

### 4.2. Preparation and Elemental Analyses of DSW of Hardness 1000 ppm

DSW was passed through the reverse osmotic sea-water desalination equipment (sdfriend Co., Ltd., Qingdao, China) and the brine and desalinated water were separated based on the principle of reverse osmosis. Then, we concentrated the brine by 100 fold using a rotary evaporator to obtain a concentration with a mineral ratio of Mg:Ca:Na = 3:1:1, thereafter, the concentration and the desalinated water were mixed to prepare DSW of hardness 1000 ppm [11]. The hardness value was calculated according to the following equation: Hardness = Mg (mg·L^−1^) × 4.1 + Ca (mg·L^−1^) × 2.5. Elemental analyses of DSW were performed using inductively coupled plasma mass spectrometry (ICP-MS, Agilent 7500a, Agilent Technologies, Palo Alto, CA, USA). The emission intensity measurements were made under the following conditions: RF Power 1200 W, nebulizer flow 15 L·min^−1^, and auxiliary gas 1.0 L·min^−1^. 

### 4.3. Preparation and Physicochemical Property Analyses of FPS

Fucoidans were extracted with hot water from algae *Sargassum pallidum* and further purified by fractional precipitation with ethanol. Then physicochemical properties were analyzed: the monosaccharide compositions were analyzed using HPLC, and the contents of total carbohydrates and the sulfate group were measured by phenol-vitriolic colorimetry and ion chromatography (IC), respectively [45].

### 4.4. Cell Culture and Drug Administration

HepG2, a liver cell line derived from a human hepatoblastoma, was chosen for the assay. HepG2 cells were obtained from the China Center for Type Culture Collection (CCTCC) and grown in DMEM supplemented with 10% FBS, 100 U/mL penicillin, 100 µg/mL streptomycin, and 5.5 mmol·L^−1^ d-glucose at 37 °C in a humid environment containing 5% CO_2_. Once the monolayers had become approximately 80% confluent, the cells were dissociated and seeded in plates at a density of 5 × 10^4^ cell·mL^−1^. To examine the effect of DSW and FPS, DSW of hardness 1000 ppm was administrated as dissolving medium of DMEM powder to prepare the culture medium, replacing fully distilled water to keep its working concentration at 1000 ppm, which has been used in a great deal of researches and showed good biological activities. The DMEM medium prepared using distilled water was used as the control medium; FPS were separately added to the medium at indicated concentrations, and for dose–response experiments, FPS of different concentrations (5, 10, 50, 100, and 500 mg·L^−1^) were used. Besides, metformin (Mef) at 1 mmol·L^−1^ was used as the positive control.

### 4.5. Cytotoxity Assay 

HepG2 cells were seeded in 96-well plates and incubated with DSW of hardness 1000 ppm and FPS of different concentrations (5, 10, 50, 100, and 500 mg·L^−1^) for 24 h. Thereafter, the medium was changed and cells were incubated with MTT (0.5 mg·mL^−1^) followed by 4 h additional incubation time. Then, the supernatant was removed and 150 μL DMSO was used to dissolve the formazan crystal. The cell viability was calculated by reading the absorbance at 570 nm.

### 4.6. Establishment of IR-Hepg2 Cell Model by High Glucose Concentration

HepG2 cells were seeded in plates, and after they adhered, they were incubated in serum-free DMEM containing either a normal glucose concentration (5.5 mmol·L^−1^ d-glucose) or high glucose concentration (30 mmol·L^−1^ d-glucose) for 24 h. The cells in 30 mmol·L^−1^ d-glucose would be considered as the IR model group [46].

### 4.7. Hepatic Glucose Production Assay

HepG2 cells were seeded in 96-well plates, and co-treated with a high glucose concentration and combination treatment of DSW of hardness 1000 ppm and FPS of different concentrations (5, 10, 20, and 50 mg·L^−1^) for 24 h. Thereafter, the medium was removed and cells were washed three times with PBS to remove glucose, incubated for 16 h in 1 mL of glucose production medium (glucose-and phenol red-free DMEM containing gluconeogenic substrates, 20 mmol·L^−1^ sodium lactate, and 2 mmol·L^−1^ sodium pyruvate) and in the presence of 1 nmol·L^−1^ insulin during the last 3 h. Then, medium was sampled for the measurement of glucose concentration using a glucose assay kit following the instructions. Glucose concentration was normalized to a cellular protein concentration [47].

### 4.8. Analysis of Glycogen Contents

Cells were co-treated with a high glucose concentration and DSW of hardness 1000 ppm and/or FPS for 24 h, and were then incubated for 3 h in the presence of 1 nmol·L^−1^ insulin, and glycogen content was measured by anthrone-sulfuric acid colorimetry, which was normalized with a cellular protein concentration [47].

### 4.9. Preparation of Protein Extract of HepG2 Cells

Cells were co-treated with a high glucose concentration and DSW of hardness 1000 ppm and/or FPS for 24 h, and the proteins of cells were then harvested in a cold RIPA buffer (1% NP-40, 50 mmol·L^−1^ Tris-base, 0.1% SDS, 0.5% deoxycholic acid, 150 mmol·L^−1^ NaCl, pH 7.5) containing protease and phosphatase inhibitor cocktails (leupeptin (10 μg·L^−1^) and sodium orthovanadate (10 μg·L^−1^)) for 30 min at 4 °C. All mixtures were then centrifuged at 12,000 rpm at 4 °C for 15 min, and the protein concentrations of the supernatants were determined using a BCA protein assay kit.

### 4.10. Measurement of Cellular Triglyceride

Cell lysates were prepared as mentioned above, and triglyceride contents in cell lysates were determined using a triglyceride assay kit following the instruction book and were expressed as µg of lipid/mg of cellular protein.

### 4.11. Western Blot Analysis

Cells were co-treated with a high glucose concentration and DSW of hardness 1000 ppm and/or FPS for 24 h. At the end point of treatment, cells were stimulated by 100 nmol·L^−1^ insulin for 10 min and then harvested. Equal amounts of protein samples (40 μg) were subjected to SDS-polyacrylamide gel electrophoresis, and transferred to nitrocellulose membranes. Membranes were blocked with 5% BSA and then incubated with the primary antibody at 4 °C overnight. Afterward, membranes were washed three times in TBST and incubated with the secondary antibody, and the bands were eventually visualized using an enhanced chemiluminescence (ECL) kit (Pierce Biotechnology, Rockford, IL, USA). The intensity of the bands was quantified with Image J 1.51b (National Institutes of Health, Bethesda, MD, USA).

### 4.12. Immumoprecipitation

The total cell lysate was centrifuged at 12,000 rpm for 20 min. The aliquot of supernatant (500 µg total protein) was then incubated with antibodies (5 µL) against IRS-1 in immuno-precipitation buffer and gently rocked overnight at 4 °C. The immuno-complexes were adsorbed by protein A/G beads for 2 h at 4 °C during gentle agitation and subsequently collected by centrifugation at 12,000 rpm for 30 s at 4 °C. Beads were then washed three times with ice-cold PBS, incubated for 10 min at 95 °C with 20 µL electrophoresis buffer, and the complete supernatant was used for Western blot analysis.

### 4.13. Statistical Analysis

Data were analyzed using an unpaired *t* test and represented as mean ± SD from three independent experiments. A value of *p* < 0.05 was considered statistically significant.

## 5. Conclusions

In conclusion, the present survey suggested that IR induced by high glucose concentrations in HepG2 hepatocytes could be improved by combination treatment of DSW and FPS. They synergistically enhanced the Tyr phosphorylation of IRS-1, and Ser phosphorylation of Akt and GSK-3β, which in turn decreased hepatic glucose production and enhance glycogen synthesis. Moreover, they also activated the phosphorylation of AMPK and ACC, which in turn decreased the intracellular triglyceride level. In short, they might reverse IR in HepG2 cells by targeting the Akt/GSK-3β and AMPK pathways. The study represents a primary effect and mechanism of combination treatment of DSW and FPS in attenuating an insulin resistant state.

## Figures and Tables

**Table 1:** Element contents in DSW of hardness 1000 ppm.

Element	Macro Element/mg·L^−1^			Trace Element/µg·L^−1^						
	Na	Ca	Mg	V	Cr	Mn	Zn	Se	Hg	Pb
Content	68.3	70.6	201.8	0.62	1.2	1.38	5.7	0.48	ND	ND
